# The Pathogenesis of Diabetes Mellitus by Oxidative Stress and Inflammation: Its Inhibition by Berberine

**DOI:** 10.3389/fphar.2018.00782

**Published:** 2018-07-27

**Authors:** Xueling Ma, Zhongjun Chen, Le Wang, Gesheng Wang, Zihui Wang, XiaoBo Dong, Binyu Wen, Zhichen Zhang

**Affiliations:** ^1^Beijing University of Chinese Medicine, Beijing, China; ^2^Dalian Municipal Central Hospital Affiliated of Dalian Medical University, Dalian, China; ^3^Dongfang Hospital, Beijing University of Chinese Medicine, Beijing, China; ^4^Chaoyang Hospital, Capital Medical University, Beijing, China

**Keywords:** pathogenesis, diabetes mellitus, oxidative stress, inflammation, cytokines, signaling pathways

## Abstract

A substantial knowledge on the pathogenesis of diabetes mellitus (DM) by oxidative stress and inflammation is available. Berberine is a biologically active botanical that can combat oxidative stress and inflammation and thus ameliorate DM, especially type 2 DM. This article describes the potential of berberine against oxidative stress and inflammation with special emphasis on its mechanistic aspects. In diabetic animal studies, the modified levels of proinflammatory cytokines and oxidative stress markers were observed after administering berberine. In renal, fat, hepatic, pancreatic and several others tissues, berberine-mediated suppression of oxidative stress and inflammation was noted. Berberine acted against oxidative stress and inflammation through a very complex mechanism consisting of several kinases and signaling pathways involving various factors, including NF-κB (nuclear factor-κB) and AMPK (AMP-activated protein kinases). Moreover, MAPKs (mitogen-activated protein kinases) and Nrf2 (nuclear factor erythroid-2 related factor 2) also have mechanistic involvement in oxidative stress and inflammation. In spite of above advancements, the mechanistic aspects of the inhibitory role of berberine against oxidative stress and inflammation in diabetes mellitus still necessitate additional molecular studies. These studies will be useful to examine the new prospects of natural moieties against DM.

## Background

Diabetes mellitus, especially type 2 diabetes mellitus (T2DM), is a very distressing pathology throughout the world. In spite of the extensive research, the exact mode of the pathogenesis of T2DM is still unclear. Therefore, the investigators are actively attempting to explore the pathogenesis of T2DM, particularly development of T2DM through oxidative stress and inflammation (Evans et al., 2005; Donath and Shoelson, 2011; Mazidi et al., 2017).

The metabolic disorders could lead to oxidative stress, which harmfully affects the insulin activity (Bonnefont-Rousselot, 2002; Furukawa et al., 2004) through several interacting pathways (Alberici et al., 2011) and generating the reactive oxygen species (ROS) such as hydrogen peroxide and superoxide anions (Rosen et al., 2001). These species could deteriorate the islets β-cells of the pancreas resulting in the reduced release of insulin (Evans et al., 2003). Besides, several signaling pathways in cells, for instance, NF-κB (nuclear factor-κB) and PKC (protein kinase C), could also be activated by ROS. It could lead to interference with the insulin signaling pathways resulting in the development of insulin resistance (IR) (Scivittaro et al., 2000; Kaneto et al., 2002; Goldin et al., 2006).

One of the crucial risk factors of DM is inflammation (Donath and Shoelson, 2011; Xie and Du, 2011). The inflammatory condition triggers the development of IR and DM through a very complex mechanism consisting of several kinases and signaling pathways (Crook, 2004; Donath, 2013; Mahmoud and Al-Ozairi, 2013; Patel et al., 2013; Gratas-Delamarche et al., 2014). Mechanistically, the adipocytes and immunocytes produce various proinflammatory cytokines including IL-6 (interleukin-6) and TNF-α (tumor necrosis factor-α) that are involved in the pathogenesis of DM (Crook, 2004; Donath, 2013; Mahmoud and Al-Ozairi, 2013; Patel et al., 2013; Gratas-Delamarche et al., 2014). These cytokines are involved in the activation of the NF-κB pathway leading to serine phosphorylation of IRS (insulin receptor substrate) resulting in the IR (Mahmoud and Al-Ozairi, 2013; Patel et al., 2013). Additionally, DM is also induced by the islets β-cells dysfunctioning, caused by excessive IL-6 and TNF-α (Donath, 2013).

**Figure 1:**
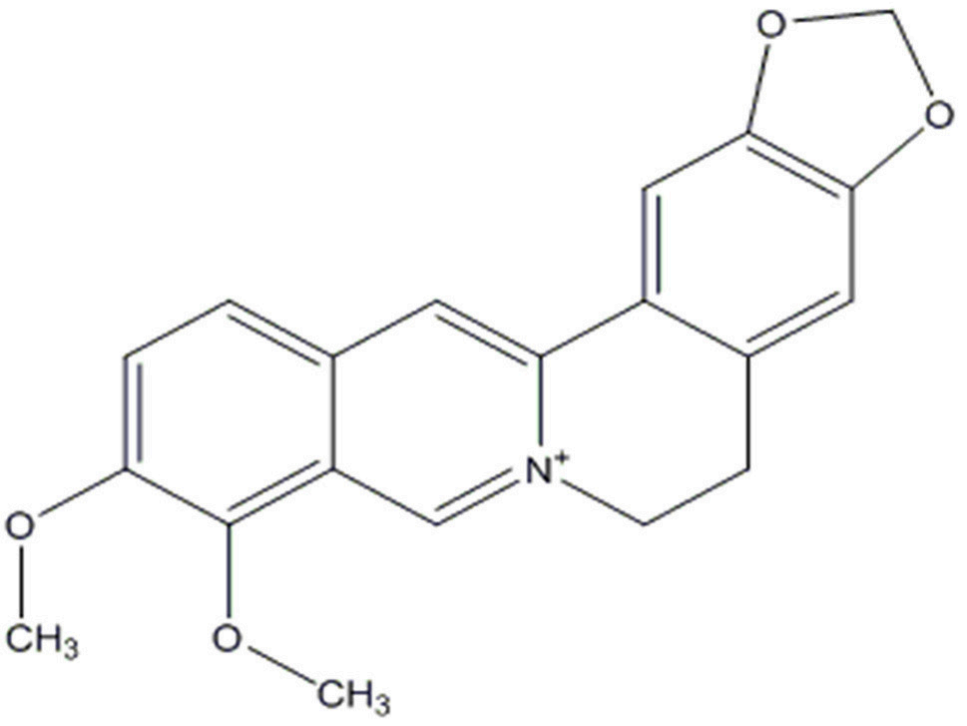
Chemical structure of berberine (Dong et al., 2012).

**Figure 2:**
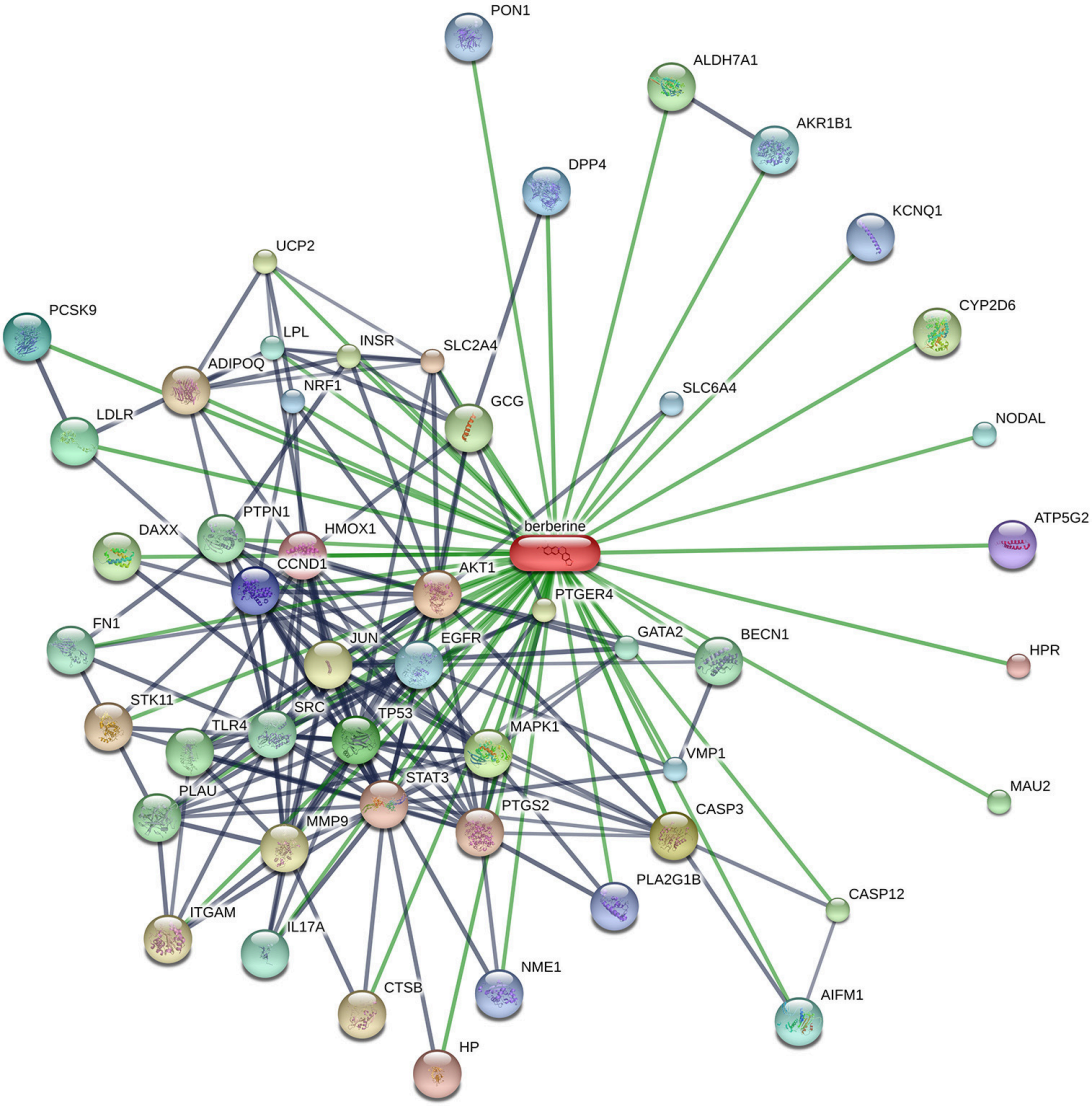
Schematic illustration of the protein networks of berberine and its interacting entities, acquired from STITCH database (accessed in March, 2017). Thicker lines represent the stronger linkages. Gray and green lines show the protein-protein interaction. [AKT1, v-akt murine thymoma viral oncogene homolog 1; CASP3, caspase 3; MAPK1, mitogen-activated protein kinase 1; TP53, tumor protein p53; LDLR, low density lipoprotein receptor; PCSK9, proprotein convertase subtilisin/kexin type 9; DPP4, dipeptidyl-peptidase 4; CCND1, cyclin D1; ATP5G2, ATP synthase; HMOX1, heme oxygenase (decycling) 1; HPR, haptoglobin-related protein; HP, Haptoglobin; STAT3, signal transducer and activator of transcription 3; PTGS2, prostaglandin-endoperoxide synthase 2; SLC2A4, solute carrier family 2 (facilitated glucose transporter), member 4; STK11, serine/threonine kinase 11; ADIPOQ, Adiponectin; CTSB, cathepsin B; ITGAM, integrin, alpha M; MMP9, matrix metallopeptidase 9; JUN, jun proto-oncogene; PTGER4, prostaglandin E receptor 4; UCP2, uncoupling protein 2; INSR, insulin receptor; CYP2D6, cytochrome P450 enzyme; GCG, glucagon; DAXX, death-domain associated protein; CASP12, caspase 12 (gene/pseudogene); ALDH7A1, aldehyde dehydrogenase 7 family, member A1; MAU2, MAU2 chromatid cohesion factor homolog; TLR4, toll-like receptor 4; PLAU, plasminogen activator, urokinase; PTPN1, protein tyrosine phosphatase, non-receptor type 1; BECN1, beclin 1, autophagy related; SRC, v-src sarcoma (Schmidt-Ruppin A-2) viral oncogene homolog (avian); FN1, fibronectin 1; GATA2, GATA binding protein 2; IL17A, interleukin 17A; LPL, lipoprotein lipase; AIFM1, apoptosis-inducing factor, mitochondrion-associated, 1; NODAL, nodal homolog; AKR1B1, aldo-keto reductase family 1, member B1 (aldose reductase); EGFR, epidermal growth factor receptor; VMP1, vacuole membrane protein 1; SLC6A4, solute carrier family 6 (neurotransmitter transporter, serotonin), member 4; NRF1, nuclear respiratory factor 1; PON1, paraoxonase 1; KCNQ1, potassium voltage-gated channel, KQT-like subfamily, member 1; and NME1, NME/NM23 nucleoside diphosphate kinase 1].

Many therapeutic moieties, both chemical and natural, are available for the management of T2DM (Yin et al., 2008; Hung et al., 2012; Patti et al., 2018). Berberine (Figure 1) is a bioactive botanical originated from *Hydrastis canadensis* and *Coptis chinensis*. It is an alkaloidal compound having a wide range of pharmacological activities (Yao et al., 2015; Zhang et al., 2015; Caliceti et al., 2016; Cicero and Baggioni, 2016; Imenshahidi and Hosseinzadeh, 2016; Cicero et al., 2017), due to its interaction with multiple proteins in the body (Figure 2). The animal and clinical studies have suggested the potential role of berberine in altering lipometabolism (Dong et al., 2012; Liu et al., 2013) and glycometabolism (Banach et al., 2018). Its major metabolites are berberrubine, thalifendine, demethyleneberberine, and jatrorrhizine (Dong et al., 2012, 2016; Yin et al., 2012; Zhang and Chen, 2012; Liu et al., 2013; Chang et al., 2016).

Owing to the excellent antidiabetic features (Pirillo and Catapano, 2015), the treatment efficacy of berberine has been found comparable with the reference antidiabetic drugs such as metformin (Zhang et al., 2010a, 2014; Liu et al., 2014, 2015; Xu et al., 2014; Goguet-Rubio et al., 2016). Berberine is also effective in combating diabetes-related pathologies (Lee et al., 2010) such as endothelial dysfunction, retinopathy (Tasdelen et al., 2013; Chang et al., 2015), nephropathy (Fu et al., 2016; Tang et al., 2016), and neuropathy (Ni et al., 2015). Based on the low toxicity and excellent efficacy of berberine (Zhang et al., 2010b; Ma et al., 2016), it has been suggested to be prescribed in the hepatic patients (Liu et al., 2013).

The ameliorated insulin sensitivity and the decrease in blood glucose level after administering berberine are attributed to the gut-microbiota modulation, islets β-cell regulation, activated AMPK (AMP-activated protein kinase), suppressed mitochondrial functions, and the upregulated insulin receptor expression (Hasan et al., 2015; Jiang et al., 2015; Lan et al., 2015; Suman et al., 2016). Current studies have reported the significance of berberine against oxidative stress and inflammation in cells, elaborating its vital role in DM. This review article summarizes the promising activities of berberine against oxidative stress and inflammation with special emphasis on its mechanistic aspects in the treatment of DM and IR.

## Antioxidant potential of berberine and the underlying mechanisms in DM treatment

### Effect of berberine on oxidative stress

Several studies (Table 1) have been conducted on animal models (Tang et al., 2006; Liu et al., 2008a, 2015; Zhou et al., 2009; Wang et al., 2011a; Zhou and Zhou, 2011; Lao-Ong et al., 2012; Wu et al., 2012; Chatuphonprasert et al., 2013; Xie et al., 2013a; Pang et al., 2015) and the cultured cells grown on high glucose-containing media to explore the antidiabetic effect pf berberine (Bhutada et al., 2011). These studies reveal that berberine possesses antioxidant feature since it promisingly inhibits oxidative stress, as evident from the altered levels of antioxidant enzymes and oxidative stress markers such as GSH (glutathione, a lipid oxidation product that is reduced in oxidative stress) and MDA (malondialdehyde that is increased in oxidative stress). Oxidative stress is characterized by lower levels of GSH (Moghaddam et al., 2013) but higher levels of MDA (Liu et al., 2008a). GSH possesses the antioxidant characteristics since it acts as a substrate of superoxide dismutase (SOD) and GSH-Px (glutathione peroxidase) enzymes and is involved in the process of peroxides scavenging (Moghaddam et al., 2013). First-line defense includes the antioxidant system that is involved in the maintenance of redox potential in the body. The performance of these antioxidants could be damaged in DM (Ceballos-Picot et al., 1996).

**Table 1:** The influence of orally administered berberine on the antioxidant parameters in diabetic rat/mice.

**Diabetes-induced animal**	**Diabetes-inducing chemical**	**Dose of berberine (mg/kg/day)**	**Treatment period (weeks)**	**Specimen used**	**Important findings**	**References**
Wistar rats	Streptozotocin	200	12	Serum	MDA*, SOD^**^	Liu et al., 2015
SD rats	Streptozotocin	200	12	Serum	MDA^*^, SOD^**^	Pang et al., 2015
Wistar rats	Streptozotocin + High fat diet	75, 150, 300	16	Serum and liver	MDA^*^, GSH^**^, SOD ^**^, GSH-Px^**^	Pang et al., 2015
Mice	Streptozotocin	200	2	Liver	GSH^*^, SOD^**^, GSH-Px^*^	Xie et al., 2013a
SD rats	Streptozotocin + High fat diet	50, 100, 150	6	Liver	Not given	Zhou and Zhou, 2011
ICR mice	Streptozotocin + Nicotinamide	100	2	Liver and kidney	GSH^*^, SOD^**^	Lao-Ong et al., 2012
SD rats	Streptozotocin + high fat diet	100, 200	8	Kidney	MDA^*^, SOD^**^	Wang et al., 2011a
Wistar rats	Streptozotocin + High fat diet	75, 150, 300	16	Pancreas	MDA^*^, SOD^**^	Chatuphonprasert et al., 2013
Wistar rats	Alloxan + High fat diet	100, 200	21	Heart	MDA^*^, SOD^**^, GSH-Px^**^	Wu et al., 2012
Wistar rats	Streptozotocin	25, 50, 100	4	Cortex and hippocampus	MDA^*^, GSH^**^	Zhou et al., 2009
Wistar rats	Streptozotocin	50, 100	8	Hippocampus	MDA^*^, SOD^**^	Tang et al., 2006

*Where*,

* and

** *signs represent decline and increase, respectively. In addition, MDA, SOD, GSH, and GSH-Px stands for malondialdehyde, superoxide dismutase, glutathione and glutathione peroxidase*.

A large number of studies (Tang et al., 2006; Liu et al., 2008b, 2015; Zhou et al., 2009; Wang et al., 2011b; Zhou and Zhou, 2011; Lao-Ong et al., 2012; Wu et al., 2012; Chatuphonprasert et al., 2013; Xie et al., 2013b; Pang et al., 2015) have supported the antioxidant activity of berberine in the model animals with alloxan- or streptozotocin-triggered hyperglycemia (Table 1). In these studies, the modified levels of antioxidant enzymes and oxidative stress markers were used as the indicators of antioxidant potential of berberine. Overall, an elevated level of GSH, GSHPx, and SOD while the suppressed level of MDA has been observed in hyperglycemic animals fed on berberine. It combats oxidative stress via scavenging the needless free radicals (Ceballos-Picot et al., 1996; Liu et al., 2008b; Moghaddam et al., 2013). One of the studies (Xie et al., 2013b) reported that mice with streptozotocin-triggered hyperglycemia showed higher levels of GSH and GSHPx and reduced contents of SOD, likely owing to the development of oxidative stress (Maritim et al., 2003; Del Rio et al., 2005; Majithiya and Balaraman, 2005). On treating these diabetic mice with berberine, there was suppressed level of GSH and GSHPx and, on the other hand, upregulation of mRNA content of SOD in different tissues including serum, liver, kidney, pancreas, heart, cortex, and hippocampus. Conclusively, it can be stated that berberine is involved in the regulation of GSH/GSHPx antioxidant system in diabetic patients. Moreover, these studies depict an association between the antioxidant potential of berberine and its suppressive influence on developing DM and IR.

### Antioxidant mechanisms of berberine against oxidative stress

**Figure 3:**
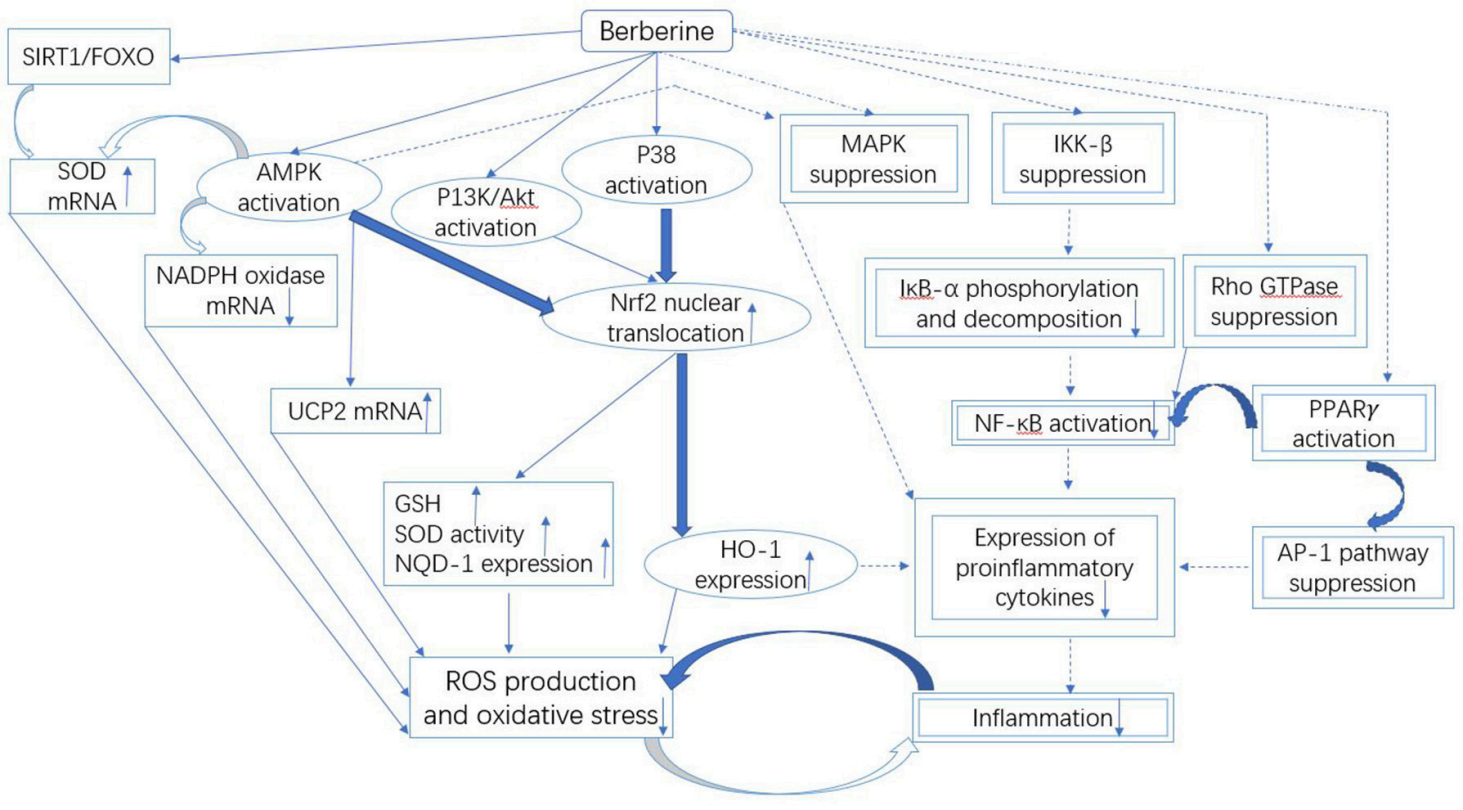
Mechanistic aspects of the antioxidant and anti-inflammatory action of berberine. It can be divided into three parts: Firstly, the downregulated NADPH oxidase expression and upregulated UCP and SOD could be involved in the berberine-induced suppression of oxidative stress that is likely controlled by the SIRT1/FOXO or AMPK pathways. Secondly, the antioxidant and anti-inflammatory action of berberine involves the activation of Nrf2 pathway, which further depends on the activated P38, AMPK and P13K/Akt signaling pathways. Finally, the inflammation is inhibited by berberine through the suppressed MAPK, Rho GTPase, NF-κB, and AP-1 pathways. The molecules and the pathways involved in the antioxidant activity of berberine are shown by the squared boxes/lines, while double squared boxes/dotted lines represent molecular species and pathways engaged in the anti-inflammatory activity. Additionally, the molecules and pathways shared by both antioxidant and anti-inflammatory activities of berberine are indicated by the encircled boxes. The pathways and the mechanisms that necessitate further investigations are shown by the curved bold lines. Berberine could terminate the malicious association between oxidative stress and inflammation.

The available data on berberine has revealed the useful relationships between oxidative stress and different cellular pathways, as illustrated in Figure 3. In literature, an *in vitro* study conducted in alkaline DMSO (dimethyl sulfoxide) has revealed the scavenging of superoxide free radicals by berberine (Hill et al., 2000). In addition, berberine-mediated upregulation of mRNA content of SOD in diabetic mice also suppresses oxidative stress (Lao-Ong et al., 2012; Xie et al., 2013b). Sirtuin 1 (SIRT1) is a deacetylase that exhibits excellent antioxidant property. While, SIRT1 triggers deacetylation of FOXO (forkhead box O) factors as well as provokes transcription of FOXO target genes including SOD in oxidative stress (Hill et al., 2000). On the other hand, the expression level of SIRT1 is reported to be augmented under the effect of berberine (Mulder et al., 1996). Thus it can be extracted from this knowledge that SIRT1/FOXO pathway is involved in the berberine-mediated increase in SOD expression.

Oxidative stress is reduced by berberine through the inhibition of expression of nicotinamide adenine dinucleotide phosphate (NADPH) oxidase also, which is a key origin of ROS (Shirwaikar et al., 2006; Zhu et al., 2013). The reason of this excessive generation of ROS could be NADPH oxidase-induced upregulation of high contents of various glycation products, fatty acids and glucose (Bonnefont-Rousselot, 2002; Furukawa et al., 2004; Xie et al., 2011). NADPH oxidase exists in different isoforms. NADPH oxidase 2/4 is the only isoform that is suppressed by berberine leading to the reduced generation of ROS (Shirwaikar et al., 2006; Zhu et al., 2013).

The onset of diabetes is related to activation of NADPH oxidase (Bonnefont-Rousselot, 2002; Furukawa et al., 2004; Xie et al., 2011), which is, therefore, a promising target for treating diabetes and comorbidities (Booth et al., 2016) such as nephropathy and neuropathy (van der Horst et al., 2004; Salminen et al., 2013). The inhibition of NADPH oxidase by berberine could suppress ROS production leading to the ameliorated effect on the diabetic condition (Zhu et al., 2013; Chang et al., 2016). On the other hand, the regulation of NADPH oxidase is negatively affected by activation of AMPK (AMP-activated protein kinase) (Sarna et al., 2010; Cheng et al., 2013), thus AMPK could be involved in the mechanism of berberine action against diabetes (Dong et al., 2016). However, it is not clear whether downregulation of NADPH oxidase by berberine occur through the activation of AMPK or some direct evidence is involved.

It is obvious from the literature that AMPK is involved in the berberine-mediated antioxidant activity.

The investigators who administered berberine to diabetic mice reported that the activation of AMPK was not only associated with downregulation of NADPH oxidase (Zhou and Zhou, 2011; Lao-Ong et al., 2012), but also related to the upregulation of SOD expression (Eid et al., 2010; Gray et al., 2013). Additionally, the expression of UCP2 (uncoupling protein 2) in arteries could be increased by berberine that, on the other hand, could suppress the arterial oxidative stress through AMPK (Eid et al., 2010). UCP2 exists in the mitochondrial membrane and is involved in the negative regulation of ROS generation and oxidative stress (Kukidome et al., 2006; Wang et al., 2010a). The contradicting information on the role of berberine in UCP2 expression, i.e. the expression of UCP2 in liver cells could be decreased by berberine has been also documented (Xie et al., 2008). Until now, it is not clear whether the regulation of UCP2 by berberine is affected by the nature of tissue or not.

It has been elaborated that the upregulation of UCP2 is dually associated with DM, i.e. the upregulated UCP2 could suppress the production of ROS in renal or adipose tissues resulting in the ameliorated diabetic condition, while in islets β cells, the upregulated UCP2 could inhibit the secretion of insulin (Wang et al., 2011b). Thus, the mechanism of berberine in the regulation of UCP2 in islets β cells needs to be revealed.

Berberine-induced suppression of oxidative stress is also mediated through Nrf2 (nuclear factor erythroid-2 related factor 2) pathway (Negre-Salvayre et al., 1997; Arsenijevic et al., 2000; de Souza et al., 2011; Yang et al., 2011). Nrf2 is a transcription factor that exhibits excellent antioxidant property via expression of HO-1 (heme oxygenase-1) and NQO-1 (NADPH quinine oxidoreductase 1) (Mo et al., 2013). Nrf2 is involved in the energy metabolism and maintenance of redox potential in cells (Mo et al., 2013). Few studies have revealed that antioxidant activity of berberine could be eliminated through the blockage of Nrf2 in neurons and macrophages (Negre-Salvayre et al., 1997; Arsenijevic et al., 2000; de Souza et al., 2011; Yang et al., 2011), thus the reduction in oxidative stress by berberine could be associated with Nrf2. Nrf2-mediated activity of berberine depends on the activation of P38, AMPK, and PI3K (phosphatidylinositol 3 kinase)/Akt pathways (Figure 3), since the blockage of these pathways could suppress the stimulating effect of berberine on Nrf2 (Negre-Salvayre et al., 1997; Arsenijevic et al., 2000; de Souza et al., 2011; Yang et al., 2011). These pathways could be activated by berberine, it leads to translocation of Nrf2 in the nucleus resulting in the activation of expression of antioxidant enzymes. It causes an increase in the cellular level of GSH and SOD that eventually suppresses the generation of ROS leading to reduced oxidative stress (Figure 3).

## The anti-inflammatory potential of berberine and the underlying mechanisms in DM treatment

### Effect of berberine on inflammation

Multiple studies (Table 2) have been conducted *in vitro* and *in vivo* to explore the anti-inflammatory effect of berberine using acute phase proteins and proinflammatory cytokines as markers (Choi et al., 2006; Jeong et al., 2009; Shang et al., 2010; Chen et al., 2011; Lin and Lin, 2011; Lou et al., 2011; Hsu et al., 2012, 2013; Bae et al., 2013; Wang, 2013; Pang et al., 2015). It has been reported that there is suppressed generation of MMP9 (matrix metalloprotease 9), TNF-α, COX2 (cyclooxygenase-2), iNOS (inducible nitric oxide synthase), MCP1 (monocyte chemoattractant protein 1), IL-6, IL-1β, CRP (C-reactive protein), and HP (hepatoglobin) in berberine-treated immune cells, hepatocytes, adipose tissues, islets β-cells or spleen cells (Choi et al., 2006; Lou et al., 2011; Hsu et al., 2012, 2013; Bae et al., 2013). One of these studies (Hsu et al., 2013) conducted on insulin resistant HepG2 cells reported the relationship between the insulin sensitizing effect of berberine and its anti-inflammatory effect. After treatment with berberine, there was a significant decline in cytokine generation and serine phosphorylation, while an increase in tyrosine phosphorylation of IRS mediated through insulin was observed in palmitate-treated HepG2 cells (Hsu et al., 2013).

**Table 2:** The influence of orally administered berberine on the proinflammatory parameters in the cultured cells or diabetic animals.

**Cultured cells or diabetic animals**	**Diabetes-inducing chemical**	**Dose of berberine**	**Treatment period (days)**	**Specimen used**	**Important findings**	**References**
Adipocytes (3T3-L1)	Not used	10 μM	0.75	3T3-L1 adipocytes	Decline in TNF-α, IL-6, CRP and HP mRNAs	Hsu et al., 2012
HepG2 cells	Palmitate	0.1–10 μM	1	Culture media	Decline in TNF-α and IL-6	Hsu et al., 2013
Macrophages (RAW 264.7)	Lipopolysaccharide	5 μM	0.25	RAW 264.7 macrophages	Decline in IL-1β and IL-6, MMP9, COX2, and iNOS mRNAs	Bae et al., 2013
Spleen cells	Lipopolysaccharide	0.8–3.3 μM	2	Culture media	Decline in TNF-α and IL-6 level	Choi et al., 2006
NIT-1 pancreatic β-cells	Lipopolysaccharide	1.25–5 μM	1	Culture media	Decline in TNF-α, IL-6, and MCP-1 level	Lou et al., 2011
KM mice	High fat diet	50 or 150 mg/kg/d	14	Serum	Decline in TNF-α and IL-6 level	Jeong et al., 2009
Wistar rats	Streptozotocin	100 mg/kg/d	42	Serum	Decline in CRP	Lin and Lin, 2011
Wistar rats	High fat diet	187.5 mg/kg/d	28	Liver	Decline in the inflammatory cell infiltration	Wang, 2013
Mice	Not used	5 mg/kg/d	28	White adipose tissue	Decline in TNF-α, IL-1β, IL-6, MCP-1, iNOS, and COX2 mRNAs	Vomhof-Dekrey and Picklo Sr, 2012
SD rats	Streptozotocin	200 mg/kg/d	84	Kidney	Decline in ICAM-1 and TGF-β1 protein expression	Pang et al., 2015
NOD mice	Not used	200 mg/kg/d	14	Splenocytes, CD4^+^ T cells	Decline in TNF-α, IL-6, IFNγ and IL-17	Shang et al., 2010
NOD mice	Not used	50, 150, 500 mg/kg/d	98	Splenocytes, Kidney and liver	Increase in IL-10/IL-1β and IL-10/IL-6 ratios, Decline in IFNγ, Increase in IL-10/IL-6 and IL-10/TNF-α ratios of mRNA levels	Chen et al., 2011

*In addition, KM mice, TNF-α, IL, CRP, HP, MMP9, COX2, iNOS, MCP-1, ICAM-1, TGF-β1, and IFNγ stands for Kunming mice, tumor necrosis factor-α, interleukin, C-reaction protein, haptoglobin, matrix metalloprotease 9, cyclooxygenase-2, inducible nitric oxide synthase, monocyte chemoattractant protein 1, intercellular adhesion molecule-1, transforming growth factor-β1 and interferon-γ, respectively*.

In other studies (Choi et al., 2006; Lou et al., 2011; Bae et al., 2013; Pang et al., 2015), after injecting various chemicals such as HFD, alloxan or streptozotocin to animals for inducing DM or IR, the reduction in the level of proinflammatory cytokines and acute phase proteins in renal, hepatic, adipose and other tissues of berberine-treated animals (Choi et al., 2006; Lou et al., 2011; Bae et al., 2013; Pang et al., 2015) was observed (Table 2). Conclusively, this decrease in inflammation was attributed to improvement in diabetic condition and its complications.

A couple of other studies (Cui et al., 2009; Xing et al., 2011) reported the decrease in inflammation, leading to the improvement in type I diabetic condition in berberine-treated NOD mice (Table 2) that exhibits the suppressed levels of various pro-inflammatory cytokines such as IL-6, IL-17, TNF-α, and IFN-γ (interferon-γ) (Cui et al., 2009; Tian et al., 2016). Besides, the investigators computed the ratio of antiinflammatory factor (IL-10) to each of the following pro-inflammatory factors such as TNF-α, IL-6, and IL-1β and found that all the ratios were increased in berberine-fed NOD mice (Xing et al., 2011). Moreover, berberine activity was observed in hepatic, renal, spleen, and other tissues (Xing et al., 2011; Tian et al., 2016).

A clinical study (Chueh and Lin, 2012a) to assess the effect of berberine in diabetic patients against inflammation was conducted. There was a significant reduction in the IL-6 level of serum after administering a dose of one gram of berberine per day for 3 months.

### Anti-inflammatory mechanisms of berberine

The suppression of inflammation by berberine is a complex phenomenon. It involves multiple pathways that are partially shared with antioxidant pathways (Figure 3).

AMPK is not only involved in the antioxidant effect, but also in the process of inflammation inhibition by berberine (Vomhof-Dekrey and Picklo Sr, 2012). In case of AMPK blockage, berberine could not inhibit the generation of pro-inflammatory cytokines such as COX2 and iNOS (Zhang et al., 2008). The elevated level of iNOS leads to the excessive release of nitric oxide resulting in the development of IR (Perreault and Marette, 2001). COX2 is involved in the prostaglandin synthesis (DuBois et al., 1998), while the prostaglandins mediate the pathogenesis of DM and its complications (Mima, 2013).

The inflammation activates MAPK (mitogen-activated protein kinase) pathway, which could be partially suppressed by berberine via activation of AMPK (Vomhof-Dekrey and Picklo Sr, 2012); it reduces the inflammation (Jeong et al., 2009; Jia et al., 2012; Vomhof-Dekrey and Picklo Sr, 2012; Wang et al., 2012). On the other hand, berberine activates P38 that plays a crucial role in combating oxidative stress and inflammation by berberine (de Souza et al., 2011; Lee et al., 2013). Thus, berberine possesses dual properties of MAPK signaling.

Similar conflicting findings were also observed in berberine-induced glucose metabolism mediated through P38. For instance, P38 is activated by berberine, which instead, enhances glucose uptake by L6 cells. Thus, P38 inhibitor could be used to terminate the berberine-mediated glucose metabolism (Cheng et al., 2006). Conversely, a study on the adipose cells did not show any involvement of P38 in glucose uptake under the influence of berberine (Zhou et al., 2007). It indicates that berberine could regulate MAPK. However, further studies are required to assess the interaction of MAPK with other signaling pathways and the resulting pharmacological effect of berberine.

Nrf2 is not only involved in antioxidant activity but also in anti-inflammatory activity of berberine (Figure 3) (Vomhof-Dekrey and Picklo Sr, 2012; Lee et al., 2013). In case of Nrf2 blockage, berberine could not suppress the production of pro-inflammatory cytokines in macrophages (Vomhof-Dekrey and Picklo Sr, 2012). The Nrf2-mediated activity of berberine activates P38 and AMPK pathways, which lead to translocation of Nrf2 in the nucleus, resulting in the suppression of pro-inflammatory cytokines (Lee et al., 2013).

Nrf2 drives the expression of an antioxidant (Mo et al., 2013) and anti-inflammatory enzyme, HO-1 (Lee et al., 2013), which is inducible by berberine (DuBois et al., 1998; Perreault and Marette, 2001; Zhang et al., 2008; Vomhof-Dekrey and Picklo Sr, 2012; Lee et al., 2013). In case of HO-1 blockage, berberine could not suppress the production of pro-inflammatory cytokines in macrophages (Lee et al., 2013). At present, HO-1 is known to be useful against DM and IR (Vomhof-Dekrey and Picklo Sr, 2012). Thus, the future studies could be focused on HO-1 as a valuable target to develop new promising drugs against DM.

The inflammation is also mediated via NF-κB pathway (Gratas-Delamarche et al., 2014), which could be targeted by berberine to induce the anti-inflammatory activity (Figure 3). In addition to food substances such as fatty acids and glucose (Goldin et al., 2006), the inflammatory stimuli including TNF-α (Gratas-Delamarche et al., 2014) could also activate IκB kinase-β (IKK-β) through serine phosphorylation (ser^181^) (Mercurio et al., 1997; Karin, 1999; Son et al., 2013) in NF-κB pathway. After feeding berberine, a decrease in activation of IKK-β and phosphorylation of ser181 in the adipose cells of the obese HFD-fed mice was noted (Yi et al., 2008). Moreover, IKK-β having a cysteine residue at position 179 is useful for berberine to exert inhibitory effect (Pandey et al., 2008).

IKK-β is involved in the phosphorylation and then degradation of IκB-α (inhibitory κB-α) (Mercurio et al., 1997; Karin, 1999; Son et al., 2013). Berberine could inhibit IKK-β leading to the stabilized IκB-α (Lee et al., 2007; Jia et al., 2012; Li et al., 2016). IκB-α turn restricts the nuclear transfer of NF-κB transcription factor (Lee et al., 2007; Jiang et al., 2011; Jia et al., 2012; Li et al., 2016), which induces the expression of pro-inflammatory cytokines including IL-6, iNOS, COX2, and TNF-α (Goldin et al., 2006; Wan et al., 2013; Gratas-Delamarche et al., 2014). These factors could be inhibited by berberine leading to negative regulation of the NF-κB pathway by berberine.

The Rho GTPase signaling pathway could be inhibited by berberine, mediating the suppression of kidney inflammation (Pang et al., 2015). Rho GTPase is a multifunctional protein that belongs to a big family of enzymes, small GTP binding proteins (Shi and Wei, 2013). Rho GTPase is involved in the positive regulation of NF-κB pathway (Xie et al., 2013b). Here we find a discrepancy, i.e., NF-κB pathway is regulated by berberine; on the other hand, berberine could suppress it by inhibiting Rho GTPase (Remppis et al., 2010; Pang et al., 2015). This activity of berberine was similar to its antioxidant property.

Activator protein 1 (AP-1) is another transcription factor that is involved in the anti-inflammatory activity of berberine (Ricote et al., 1998; Kuo et al., 2004; Schonthaler et al., 2011). Mechanistically, the berberine-mediated inhibition of AP-1 binding with DNA suppresses the production of pro-inflammatory cytokines such as COX2 and MCP1.

Berberine-mediated activation of PPARγ (peroxisome proliferator-activated receptor γ) is found to inhibit NF-κB and AP-1 (Delerive et al., 1999; Pasceri et al., 2000; Huang et al., 2006; Chen et al., 2008). It results in the reduced production of pro-inflammatory cytokines in the intestinal cells and macrophages (Zhou and Zhou, 2010; Feng et al., 2012) leading to the suppression of inflammation (Chen and Xie, 1986; Li et al., 2011).

## Clinical use of berberine

Various clinical studies have described the safety and effectiveness of berberine (at a dose of 0.2–10 g/day) in treating T2DM patients. Generally, a decrease in blood glucose level by 20–40% is reported in fasting patients treated with berberine alone, this effect resembles to that of rosiglitazone and metformin (Zhang et al., 2010a; Dong et al., 2012). Moreover, additive hypoglycemic effect was observed in the Italian T2DM patients when treated with berberine in combination with sulfonylureas or metformin, the standard hypoglycemic drugs (di Pierro et al., 2012). However, the safer nature of this botanical is its important feature in comparison with the synthetic drugs including rosiglitazone or metformin. For instance, the synthetic drugs are not recommended for comorbid patients having T2DM and chronic hepatitis, because it could result in further destruction of hepatic functions. In contrast, berberine is safe and effective, exerting an ameliorative effect on the hepatic function and blood glucose level (Zhang et al., 2010a). The likely modes of action of berberine are ameliorated insulin sensitivity, enhanced insulin release, PPARs-modulated regulation of glucose- and lipid-metabolism, suppressed uptake of glucose via enterocytes, modulated effect on gut microbiota, and the inhibitory effect on oxidation and inflammation (Kim et al., 2007; Kong et al., 2009; Vuddanda et al., 2010; Chueh and Lin, 2011, 2012b; Zhang et al., 2011a,b; Derosa et al., 2012; Yang and Yin, 2012; Singh and Mahajan, 2013; Wu et al., 2016). However, the above-mentioned conclusions are extracted from various short-term studies, which must be supported by the large-scale, high-quality, and long-term randomized clinical trials to validate the effect of berberine on DM and the diabetic complications and recommend its routine clinical use as an effective moiety against DM.

Additionally, several studies have revealed therapeutic effects of berberine on diabetic complications, including diabetic cardiovascular diseases, neuropathy, and nephropathy (Dorr et al., 2012; Yao et al., 2015). A course of treating T2DM and its associated cardiovascular diseases in human with berberine resulted in an improvement in the endothelial function, likely via suppressing oxidative stress on the vascular endothelium mediated by CD31+/CD42- microphages (Gu et al., 2011). From results, it was obvious that berberine enhanced the expression of Nox4 proteins, suppressed synthesis of NO, and increased the production of ROS in human umbilical vein endothelial cells. In addition, diabetic cardiomyopathy is another cardiovascular diseases that could likely be treated using berberine since berberine influences cardiomyopathy-inducing factors, such as oxidative stress, the homeostasis of glucose and lipids, and endothelial dysfunction (Zhang et al., 2011a). Moreover, nephropathy is another diabetic complication that is promisingly treated by berberine. Berberine suppressed the excretion of albumin through urine, ameliorating the ratio of kidney to body weight and reducing the fasting blood glucose level, blood creatinine, glomerular area, and blood urea nitrogen in diabetic nephropathy in rats (Li and Shah, 2003; Tang et al., 2011). Additionally, berberine could significantly ameliorate nerve conduction velocity in diabetic neuropathy in rats (Hua et al., 2001). However, there are no direct scientific evidences at present to prove the role of berberine in above-stated diabetic associated complications, necessitating the additional mechanistic studies.

## Discussion

After conducting the first study in 1986 on the antidiabetic activity of berberine in animals (Chen and Xie, 1986), this phytochemical moiety emerged as an excellent antidiabetic molecule (Ni, 1988). Later on, in 1988, the first clinical study in diabetic patients verified the blood glucose lowering potential of berberine (Lugrin et al., 2013). Hitherto, a large number of studies have been conducted to explore the molecular basis of berberine activity against diabetes and its complications. In this review article, the antioxidant and anti-inflammatory effects of berberine against DM are summarized.

Metabolically, there is a strong association between oxidative stress and inflammation (Soskic et al., 2011; Munoz and Costa, 2013; Gratas-Delamarche et al., 2014), owing to their regulation by the shared regulators such as NF-κB (Goldin et al., 2006; Soskic et al., 2011; Munoz and Costa, 2013; Gratas-Delamarche et al., 2014). Oxidative stress-induced generation of pro-inflammatory cytokines including IL-6 and TNF-α mediates the production of ROS, which enhances the oxidative stress (Zhang et al., 2012; Donath, 2013). Obviously, the malicious association between oxidative stress and inflammation could damage IR (Xie and Du, 2011; Gratas-Delamarche et al., 2014). Berberine could exert the inhibitory effect on oxidative stress and inflammation via multiple cellular pathways, for instance, AMPK signaling pathway (Vomhof-Dekrey and Picklo Sr, 2012; Lee et al., 2013). Future study can be executed to examine the restrictive effect of berberine on oxidative-inflammatory cycle.

Several mechanisms relating to berberine-mediated inhibition of oxidative stress and inflammation have been already proposed. Further to the above described modes of berberine, its activity against inflammation and gut-associated valuable effects are associated with each other (Xie and Du, 2011; Liu et al., 2016; Jia et al., 2017). Since berberine is poorly bioavailable (Peng et al., 2009; Han et al., 2011; Shan et al., 2013), the most part of its orally administered quantity could be involved in modulating colonic flora that leads to a buildup of short-chain fatty acids. These fatty acids are not only involved in the enhanced production of gut bacteria (Xie and Du, 2011), but it also plays a crucial role in the amelioration of selective permeability of intestine and suppression of inflammation by preventing entry of toxic materials into the systemic circulation (Zhang et al., 2011b). This concept has been proven by the ameliorative role of berberine in TNF-α–induced mucosal damage in the intestine (Amasheh et al., 2010; Tan et al., 2013). In spite of low oral bioavailability of berberine (Wang et al., 2012), berberine and its metabolites have been found in excess in several organs such as liver and kidney, explaining its excellent biological effects even with its low systemic contents (Peng et al., 2009; Wang et al., 2012). The metabolites of berberine have also been found to be involved in activating AMPK (Wu et al., 2013). Until now, no study has been conducted to explain the inhibitory role of berberine metabolites against oxidative stress and inflammation mediating DM and IR.

There are still several concerns about the antioxidant and anti-inflammatory role of berberine that necessitate further studies. For instance, the conflicting findings of the berberine-mediated regulation of PPARγ, UCP2, and MAPKs signaling pathways are not clarified yet. Based on the inhibitory potential of berberine against oxidative stress and inflammation, treatment of DM by berberine needs to be studied in the clinical setting. Moreover, berberine has been found to effectively combat endoplasmic reticulum stress in islets β-cell impairment and IR (Xue et al., 2005; Hanada et al., 2007; Wang et al., 2010b). Endoplasmic reticulum stress has grown into an excellent therapeutic target (Evans-Molina et al., 2013) owing to its involvement in the pathogenesis of DM via ROS and inflammation (Evans-Molina et al., 2013). Future studies should be focused on studying the effect of berberine on the endoplasmic reticulum stress and its association with oxidative stress and inflammation, explaining the mechanism of berberine against DM. These studies will be valuable to explore new horizons of natural moieties against DM.

## Conclusion

The naturally existing phytochemical berberine exhibits an excellent activity against oxidative stress and inflammation via several signaling pathways including NF-κB, AMPK, Nrf2/HO, and MAPKs pathways and various kinases in cells, and likely contribute to treating DM and IR (Figure 3). Owing to the growing attention of clinicians in the usage of berberine during last two decades, the mechanistic aspects of the inhibitory role of berberine against oxidative stress and inflammation necessitate the advance studies at the molecular level.

## Author contributions

XM, ZC, LW, and ZZ designed and wrote this review, and GW, ZW, XD, and BW collected the information.

### Conflict of interest statement

The authors declare that the research was conducted in the absence of any commercial or financial relationships that could be construed as a potential conflict of interest.

## Abbreviations

DM: diabetes mellitus
NF-κB: nuclear factor-κB
AMPK: AMP-activated protein kinases
MAPKs: mitogen-activated protein kinases
Nrf2: nuclear factor erythroid-2 related factor 2
ROS: reactive oxygen species
PKC: protein kinase C
IR: insulin resistance
IL-6: interleukin-6
TNF-α: tumor necrosis factor-α
IRS: insulin receptor substrate
AKT1: v-akt murine thymoma viral oncogene homolog 1
CASP3: caspase 3
MAPK1: mitogen-activated protein kinase 1
TP53: tumor protein p53
LDLR: low density lipoprotein receptor
PCSK9: proprotein convertase subtilisin/kexin type 9
DPP4: dipeptidyl-peptidase 4
CCND1: cyclin D1
ATP5G2: ATP synthase
HMOX1: heme oxygenase (decycling) 1
HPR: haptoglobin-related protein
HP: Haptoglobin
STAT3: signal transducer and activator of transcription 3
PTGS2: prostaglandin-endoperoxide synthase 2
SLC2A4, solute carrier family 2 (facilitated glucose transporter): member 4
STK11: serine/threonine kinase 11
ADIPOQ: Adiponectin
CTSB: cathepsin B
ITGAM, integrin: alpha M
MMP9: matrix metallopeptidase 9
JUN: jun proto-oncogene
PTGER4: prostaglandin E receptor 4
UCP2: uncoupling protein 2
INSR: insulin receptor
CYP2D6: cytochrome P450 enzyme
GCG: glucagon
DAXX: death-domain associated protein
CASP12: caspase 12 (gene/pseudogene)
ALDH7A1, aldehyde dehydrogenase 7 family: member A1
MAU2: MAU2 chromatid cohesion factor homolog
TLR4: toll-like receptor 4
PLAU, plasminogen activator: urokinase
PTPN1, protein tyrosine phosphatase: non-receptor type 1
BECN1, beclin 1: autophagy related
SRC: v-src sarcoma (Schmidt-Ruppin A-2) viral oncogene homolog (avian)
FN1: fibronectin 1
GATA2: GATA binding protein 2
IL17A: interleukin 17A
LPL: lipoprotein lipase
AIFM1, apoptosis-inducing factor, mitochondrion-associated: 1
NODAL: nodal homolog
AKR1B1, aldo-keto reductase family 1: member B1 (aldose reductase)
EGFR: epidermal growth factor receptor
VMP1: vacuole membrane protein 1
SLC6A4, solute carrier family 6 (neurotransmitter transporter, serotonin): member 4
NRF1: nuclear respiratory factor 1
PON1: paraoxonase 1
KCNQ1, potassium voltage-gated channel, KQT-like subfamily: member 1
NME1: NME/NM23 nucleoside diphosphate kinase 1
GSH: glutathione
MDA: malondialdehyde
SOD: superoxide dismutase
GSH-Px: glutathione peroxidase
DMSO: dimethyl sulfoxide
SIRT1: Sirtuin 1
FOXO: forkhead box O
NADPH: nicotinamide adenine dinucleotide phosphate
HO-1: heme oxygenase-1
NQO-1: NADPH quinine oxidoreductase 1
PI3K: phosphatidylinositol 3 kinase
MMP9: matrix metalloprotease 9
COX2: cyclooxygenase-2
iNOS: inducible nitric oxide synthase
MCP1: monocyte chemoattractant protein 1
CRP: C-reactive protein
HP: hepatoglobin
IFN-α: interferon-β
IL-10: interleukin-10
IKK-β: IκB kinase-β
HFD: high fat diet
IκB-α: inhibitory κB-α
AP-1: activator protein 1
PPARγ: peroxisome proliferator-activated receptor γ.
